# Sensitive Readout for Microfluidic High-Throughput Applications using Scanning SQUID Microscopy

**DOI:** 10.1038/s41598-020-58307-w

**Published:** 2020-01-31

**Authors:** Shai Wissberg, Maria Ronen, Ziv Oren, Doron Gerber, Beena Kalisky

**Affiliations:** 1 Department of Physics, Bar-Ilan University, Ramat-Gan, 52900 Israel; 2 Institute for Nanotechnology and Advanced Materials, Bar-Ilan University, Ramat-Gan, 52900 Israel; 3 Mina and Everard Goodman Faculty of Life Sciences, Bar-Ilan University, Ramat-Gan, 52900 Israel; 4 Department of Biotechnology, Israel Institute for Biological Research, Nes-Ziona, 7410001 Israel

**Keywords:** Microfluidics, Imaging techniques, Biological physics, Lab-on-a-chip, Nanoparticles

## Abstract

Microfluidic chips provide a powerful platform for high-throughput screening of diverse biophysical systems. The most prevalent detection methods are fluorescence based. Developing new readout techniques for microfluidics focusing on quantitative information in the low signal regime is desirable. In this work, we combine the well-established immunoassay approach, with magnetic nanoparticles, with a highly sensitive magnetic imaging technique. We offer to integrate a microfluidic array into a scanning superconducting quantum interference device (SQUID) microscope, to image nanoparticles that were moved through the microfluidic device. We demonstrate the technique on protein-protein interactions (PPI). We compare sensitivity to that of a conventional readout, quantify the amount of interactions, and demonstrate 0.1 atto-mole sensitivity. Our work serves as a proof of concept that will promote the development of a new set of eyes, a stable usable microfluidic-scanning SQUID microscopy.

## Introduction

Microfluidic technologies are of growing impact on research and medicine. Their potential for high throughput screening is well established^1–4^. For example, a microfluidic platform for high-throughput screening of protein-protein interactions (PPI), called protein interaction network generator (PING), was developed to improve conventional protein microarrays^1,5^. The most common readout technique for these high throughput platforms is fluorescence. In many of these screening applications and in PPI screening platforms in particular, the sensitivity provided by the fluorescence readout is a limiting factor.

Protein microarrays are a powerful platform for high-throughput interaction screening. Different proteins are immobilized onto solid supports and the binding of interacting protein is detected in simultaneously using fluorescence labeling. One of the most challenging aspects of this technology is the functional immobilization of a large number of different proteins with different physicochemical properties. PING overcomes this hurdle^1,5^. PING combines on-chip *in vitro* protein synthesis with a microfluidic immunoassay. This platform provides a tool for measuring functional proteins, including membrane proteins. The latter are very difficult to purify and study in significant quantities, especially in their active form. In cells, they are usually in minute amount, but play a critical role in cellular functioning including transferring information back and forth across membrane barriers. This microfluidic platform is still bound by the limits of sensitivity provided by fluorescence detection. Thus, it serves as a good model for the sensitivity limitation of conventional fluorescence readout.

To date, fluorescence labeling is the prevalent labeling method for biophysical screening platforms. Fluorescence probes are available in many forms, from small organic and quantum dot molecules to fluorescent proteins^6^. They have a large linear dynamic range and fast detection speed, and also are of affordable cost. However, major limitations include problems such as photo-stability and sensitivity^7^. While some fluorescent measurements such as total internal reflection fluorescence can reach single molecule sensitivity, they are limited in their throughput and their compatibility with microfluidics^8,9^.

The importance of weak and transient interactions highly motivates the development of new sensitive detection methods that are compatible with high-throughput screening. For instance Plasmon resonance, a technique that measures molecular binding events at a metal surface by detecting changes in the local refractive index^10^; piezo resistive-based method^11^; or Electrochemical-based sensors, which vary in their different chemistries, but depend on the solid electrode surface, interactions with the target protein and the molecular recognition layer^12^. Another well-established method for PPI detection is Atomic Force Microscopy (AFM). In this method, a molecule is bonded to the edge of the AFM tip, which is then rastered over the sample. When the molecule interacts with proteins on the surface of the sample, a force is exerted over the cantilever^13^. This method uses mechanical contact to perform the detection, and therefore is less suitable for high-throughput readout for microfluidics.

Another approach for improving the sensitivity of the readout is by incorporating magnetic nanoparticles. Bio-sensing using magnetic nanoparticles has been widely used for bio-separation^14^, bio-therapeutics^15,16^, immunoassay^17,18^, and even for detecting PPI, at varying quantities^19,20^. In the latter, a Giant magneto-resistive sensors are usually applied. However, the sensitivity of these sensors is usually not sufficient to detect a single nanoparticle^21^.

Another common highly sensitive magnetic sensor is the Superconducting Quantum Interference Device (SQUID), composed of a superconducting loop with two Josephson junctions. A SQUID sensor converts magnetic flux that threads its loop to a detectable electric signal, with a period of one flux quantum^22,23^, Φ_0_. SQUIDs have been widely used in magneto-sensing of bio-magnetism in different biological systems^24–27^, including bioassays^28^. These sensors are usually sensitive enough to detect a single magnetic nanoparticle, yet to date, incorporating them as a readout for high-throughput platforms lacked in spatial resolution^29^.

In this work, we use sensitive magnetic detection to expand the limits offered by conventional fluorescence, as a readout of high throughput microfluidic platforms. We label the interactions with magnetic nanoparticles and use a highly sensitive magnetic imaging technique, to detect these nanoparticles. Here, we offer a proof of concept experiment, to demonstrate the advantage of combining scanning SQUID microscopy with a microfluidic platform, to provide improved sensitivity for high throughput screening of biophysical systems.

We hope this data will lead to the development of an integrated platform, combining scanning SQUID microscopy with microfluidics.

## Results

We utilize a SQUID sensor, which is designed for local measurements, by defining a small sensitive area, the pickup loop, that is brought close to the sample of interest^30,31^. We control the position of the sensor relative to the sample using piezoelectric elements, and generate maps of the static magnetic landscape.

**Figure 1:**
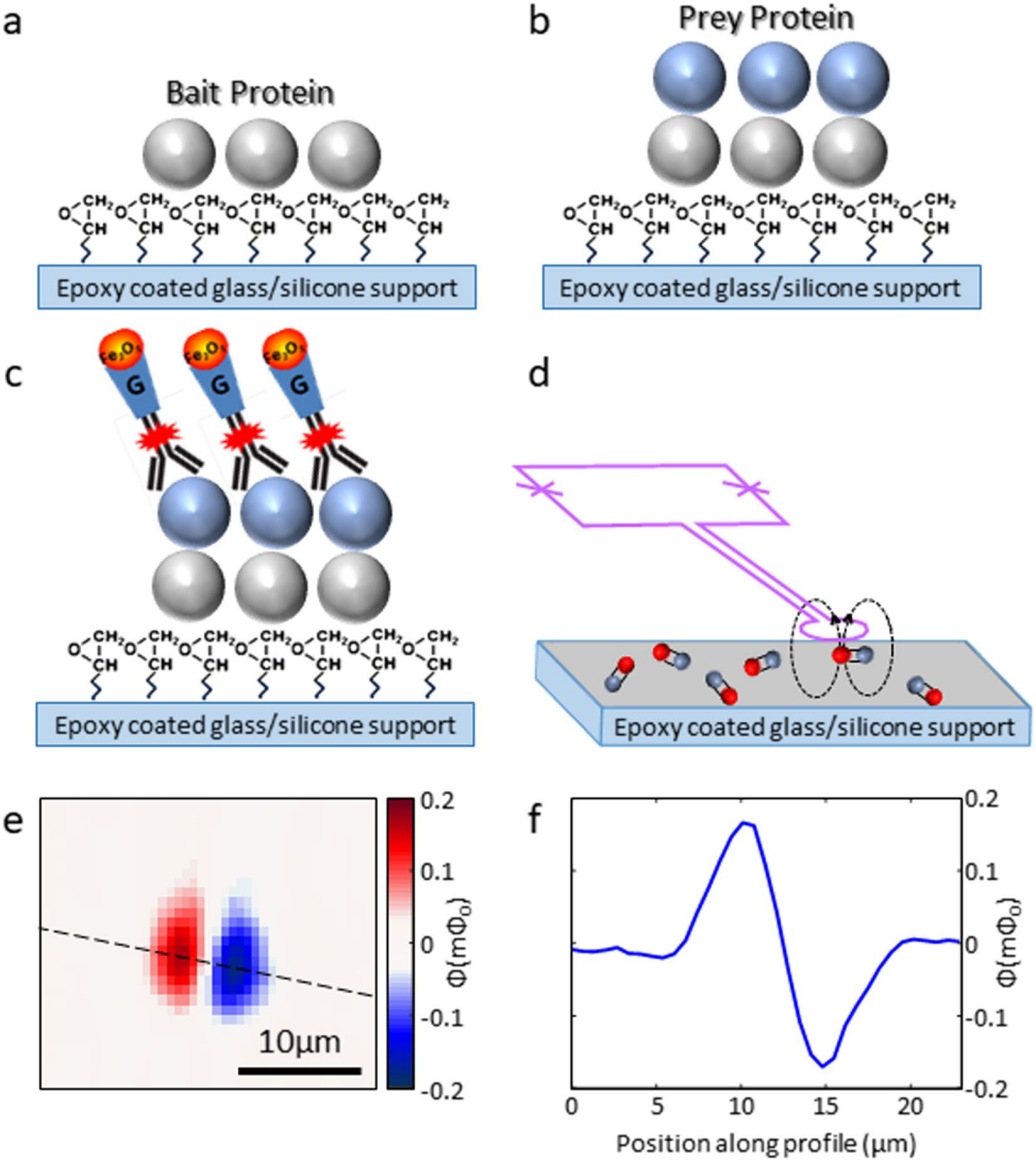
Illustration of our detection process. (**a**) A library of bait proteins is immobilized onto an epoxy coated support. (**b**) A prey protein is inserted and allowed to interact with the bait protein. (**c**) The interaction is labeled using anti-HIS Alexa flour 647 antibody conjugated to protein G magnetic nanoparticle (IPG10). (**d**) A scanning SQUID magnetometer detects PPIs. A sensing loop is extended from the SQUID loop (purple) to capture magnetic signals near the surface. Magnetic nanoparticles (red) are connected to PPIs (gray). The dashed lines describe the way magnetic field lines spread near the magnetic nanoparticle. (**e**) Magnetic image of the flux measured from a nanoparticle. The two lobes indicate an ingoing and outgoing field lines, relative to the plane of the image. (**f**) Line-cut at the dashed line in (**e**).

Figure 1 describes the experimental setup, combining the magnetic measurement with PING. The magnetic landscape imaged by the scanning SQUID contains magnetic dipoles which are generated by the nanoparticles that tag the interactions. We couple fluorescent antibody and protein G conjugated single-core iron oxide nanoparticle (Fig. 1a–c) for detecting PPI by both magnetic and fluorescent detection techniques. The high sensitivity of the scanning SQUID probe (Fig. 1d, 170 µB∙Hz^−1/2^^32^), capable of detecting individual nanoparticles^33^, has the potential to enhance our ability to detect small amount of interactions.

The capability to detect small number of magnetic nanoparticles, down to individuals, is based on the fact that the pickup loop is relatively small and is brought close enough to the particle, so that it captures the field lines before they close (see illustration in Fig. 1d). The magnetic signal we show in Fig. 1e is smeared over several micrometers because we image several micrometers away from the sample and field lines spread. The area of interest is several millimeters, which include an array of active surfaces with a diameter of 80 micrometers.

**Figure 2:**
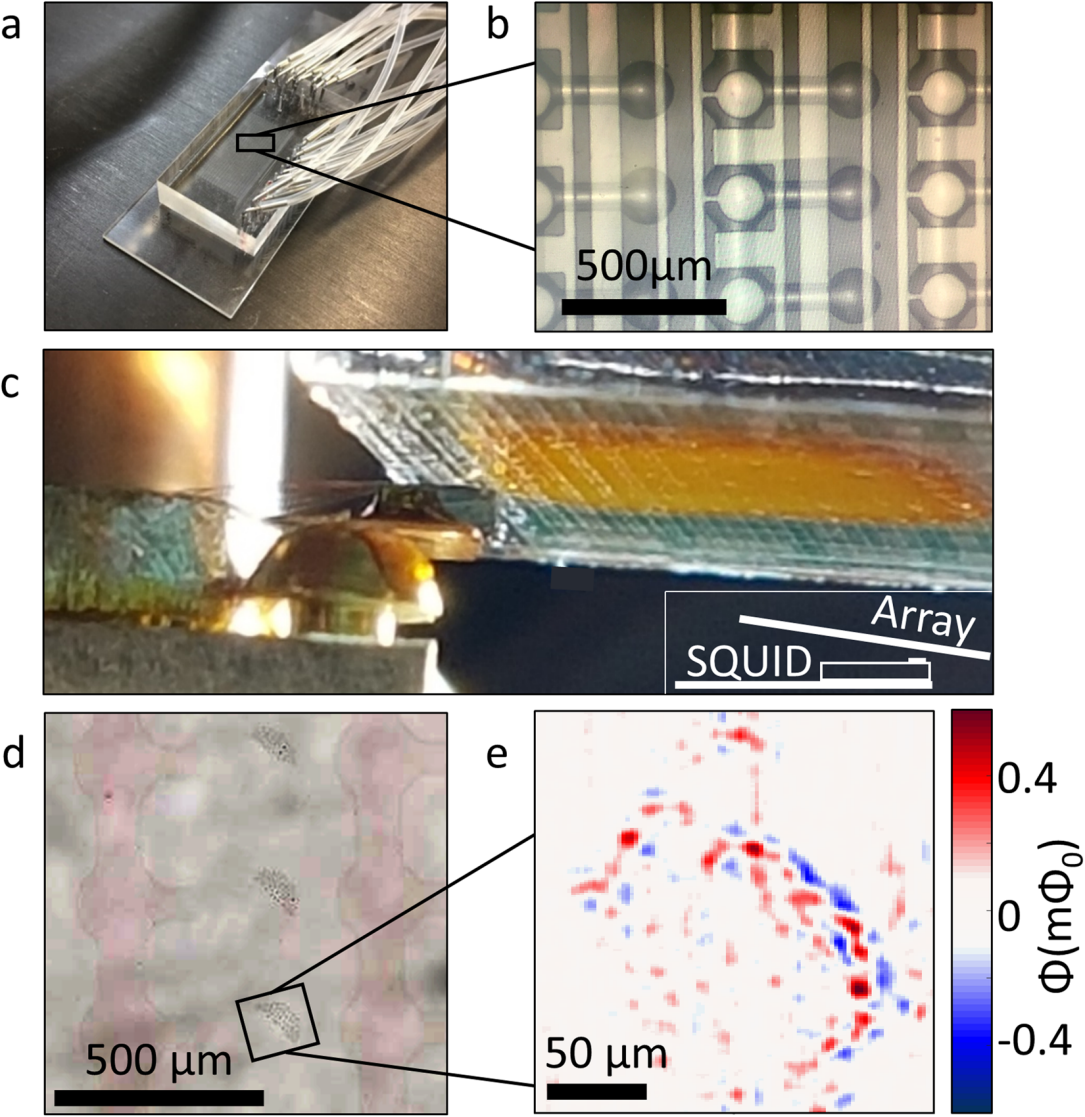
Imaging of a microfluidic device. (**a**) The microfluidic device in its initial state, with the PDMS tubes network. (**b**) The microarray on the microfluidic device. The magnetic nanoparticles are moved through the device and settle at the side chambers. (**c**) Image of the measurement configuration. The scanning SQUID sensor seats on a cantilever as it approaches the microfluidic array. Inset: A sketch depicting the alignment of the measurement. (**d**) Optical image of the solution after it dried out, and the device was peeled off, leaving the magnetic nanoparticles on the glass. (**e**) Magnetic image of nanoparticles on the glass. The concentration of the nanoparticles is visible and can be seen to vary in its intensity.

To demonstrate the potential of integrating the microfluidic chip in the scanning SQUID microscope, we moved a solution of the magnetic particles through the device^1,5^. Once the solution dried, we mounted the sample onto the scanning SQUID microscope for measurements. Figure 2a,b shows the microfluidic device. In order to achieve the desired proximity between the sensing point and the sample, we removed the Poly-Dimethyl Siloxane (PDMS) layer to expose the glass layer with the content of the channels. We then mounted the chip in the scanning microscope, and aligned the array with the scanning SQUID sensor (Fig. 2c). Figure 2d focuses on one concentration of nanoparticles, remnants from the solution that dried in a single chamber. The magnetic image of that chamber, taken by the scanning SQUID, shows a dense assembly of magnetic signals, even in locations where magnetic particles are not observed optically (Fig. 2d,e).

Next, we tested the sensitivity limit. In order to do so, we compared solutions with four different dilution factors (1:1, 1:10^2^, 1:10^4^, and 1:10^6^) of PPIs marked by magnetic nanoparticles. To avoid intermixing between the different dilutions, and for accumulating statistics, we covered different 3.5 × 5 mm^2^ SiO_2_/Si substrates with each of the dilutions. We measured the fluorescent signal from each experiment, and then imaged the magnetic signal using the scanning SQUID microscope. We recorded the magnetic fields generated by the nanoparticles as a function of location, to generate maps of the magnetic landscape.

**Figure 3:**
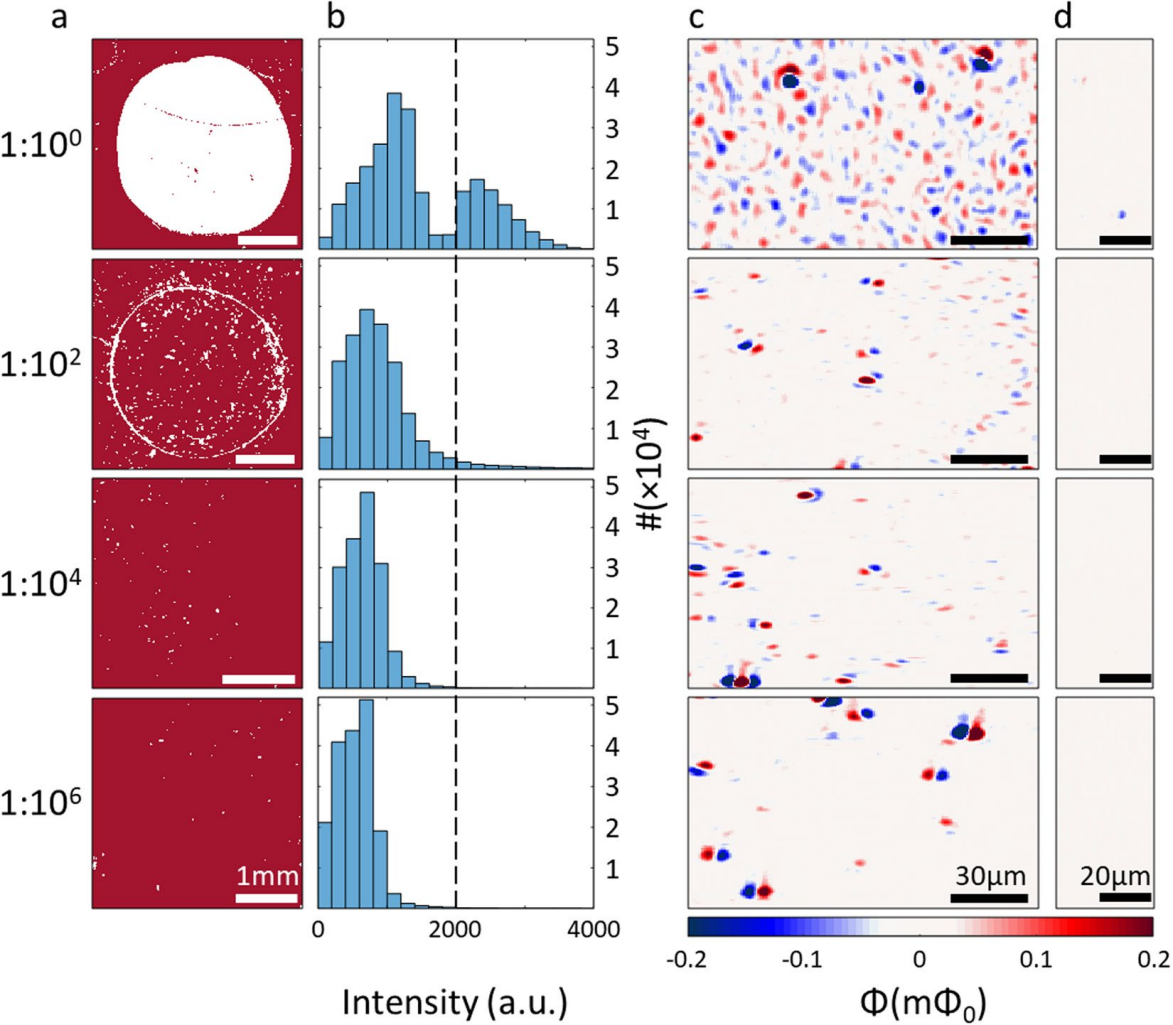
Fluorescent and magnetic signal of 4 different dilutions. (**a**) Fluorescent signal measured for each dilution. Pixels with signal higher than 1800 a.u. are shown in white, while pixels below 1800 a.u are shown in red. For the 1:1 dilution the area of interaction is visibly clear, while only the contour appears for the 1:10^2^ dilution. 1:10^4^ and 1:10^6^ dilutions show no area of interaction. (**b**) Histograms of the fluorescent measurements. For the 1:1 dilution, two peaks are visible, with the higher peak appearing above 2000 a.u. (**c**) scanning SQUID images of regions inside the interaction area. The scanning SQUID detected magnetic signals at all concentrations. In the 1:1 and 1:10^2^ dilutions, an abundance of particles are imaged, while at the higher dilutions the particles are scarcer. Few stronger dipole-looking features are visible in all images, representing locations of nanoparticle aggregates. (**d**) Regions scanned outside the area of interaction. The pixel size in these scanning SQUID images is 0.6 µm × 0.8 µm.

We compared the performance of fluorescence and scanning SQUID detection methods by measuring the interaction of Green Fluorescent Protein (eGFP), tagged with a 6X histidines at its N terminus, with anti-HIS antibody at different dilution factors. For the higher concentration, the 1:1 dilution, the fluorescent signal of the PPIs (Fig. 3a) gave a clear image of the interaction area (white circle). At a lower concentration, the 1:10^2^ dilution, we observed fluorescent signal only at the contour of the interaction area. In the more diluted samples the fluorescent signal did not detect the area of interaction. We quantified the fluorescent measurements (histogram plots, Fig. 3b.) to determine the detection cutoff. We used the two well distinguished peaks observed in the 1:1 dilution, where the fluorescent signal clearly identified the active area, to define the cutoff limit at 2000 a.u. (dashed line in Fig. 3b). As for the 1:10^2^ diluted sample only one peak is visible, but the signal intensity histogram still showed some readings corresponding to PPI (>2000 a.u.). At the higher dilutions no interaction was detected by fluorescence.

We performed magnetic imaging of the same samples and show representative magnetic maps for each dilution in Fig. 3c,d. The number of magnetic dipoles observed in each image decreased with increasing dilutions, as expected, and clearly demonstrates that even the highest dilution did not reach the sensitivity limit of the scanning SQUID. In contrast to the fluorescent reading, the scanning SQUID detected PPIs at all measured concentrations.

To quantify the number of PPIs measured by the scanning SQUID we counted the number of magnetic dipoles in each image (Table 1). Each dipole was counted as a single particle. However, some of the dipoles were much stronger than the others, representing aggregates of nanoparticles. Since we counted these aggregates as a single particle, the numbers in Table 1 serve as a lower limit to the amount of particles detected by the scanning SQUID. Please note that developing a method for PPI detection with scanning SQUID microscopy is aimed to assist detection at high dilutions, where only a small number of PPIs occur. In this regime aggregates are not expected and the ability to detect individual nanoparticles^33^ (PPIs) would be extremely beneficial.

**Table 1:** Summary of PPI measurements by scanning SQUID and fluorescence detection.

Dilution	Fluorescence measurement	Particles per mm^2^ inside the interaction area^*a,b*^	Particles per mm^2^ outside the interaction area^*b*^
1:1	Above sensing level	7,761	50
1:10^2^	At sensing level	1,772	6
1:10^4^	Below sensing level	627	2
1:10^6^	Below sensing level	279	0

^*a*^Numbers are lower limits, aggregates observed were counted as single particles.

^*b*^The areas imaged by scanning SQUID were on average of 0.9 mm^2^. For control, outside the interaction area, an average region of 0.7 mm^2^ was scanned.

We performed control experiments to determine the noise level of the scanning SQUID experiment. Although magnetic nanoparticles cannot be completely avoided in ambient conditions, they are rare. To determine the level of noise generated by random magnetic dipoles, we imaged bare SiO_2_/Si substrate. We found only one magnetic dipole on the entire scanned area (1.6 mm^2^). The singularity of this dipole demonstrates scanning SQUID capabilities as a reliable tool for preforming particle counting.

## Discussion

One of the leading drawbacks of microfluidic high throughput screening is sensitivity. For example, protein-binding methodologies suffer from low coverage and experience difficulties related to detection of low abundant proteins^34^. The second challenge of fluorescence, the common readout technique, is the detection limit at low concentrations. There are fluorescence-based methods with single molecule sensitivity, such as TIRF microscopy^9^. However, these methods can be limited by effects like photo-bleaching. This is particularly problematic when dealing with low abundant proteins in high-throughput screening. The magnetic-based scanning SQUID technique is not affected by photo-bleaching, maintaining ultra-high sensitivity. The scanning SQUID-based method we propose can complement fluorescence-based methods and improve the coverage and sensitivity.

This study provides proof of concept for the use of scanning SQUID microscopy as a potential sensitive readout for microfluidic high throughput platforms. The scanning SQUID microscope and microfluidic devices used in this study were not fully compatible. They were sufficient to answer the most basic challenge: can scanning SQUID be used as a suitable highly sensitive sensor for microfluidic platforms? The model we used for this demonstration was an immunoassay for protein interactions, using PING. We further suggest design strategies and changes required in order to create a working SQUID-microfluidic platform.

First, a simpler scanning method can be used. In the microscope we constructed for this study, the stage is controlled by s-shaped piezoelectric elements, supporting high spatial resolution, but limited to a scan area of approximately 0.3 × 0.3 mm^2^. As a result, in order to image the entire chip, we stitched together several scans. A future apparatus should integrate a different stage-moving technique, for example motor based, to allow a scan range of millimeters in order to cover the entire microfluidic chip in one image. An alternative approach is to combine an array of sensors.

Second, we used a detector with sensing loop size of 0.9 µm, which was an overkill for this study. An evolved version can accomplish the same with a simpler, larger, pickup loop. A larger loop will capture more field lines, and this would simplify the use. However, it should be noted that the pickup loop size should be kept smaller than the distance between two neighboring chambers in the microfluidic device, to avoid overlapping signals. The optimal pickup loops size should be about 10 micrometers, taking into account the trade-off between enlarging the sensing point (for quicker scans and ease of measurement), and the reduction in spatial resolution (limiting our ability to image single particles).

Third, another modification that can be made is fabricating the microfluidic device on a thin flat non-magnetic substrate (i.e. metal, glass or PDMS). The scanning SQUID sensor can sense magnetic fields from a distance^35^ or through a buffer layer as long as the object of interest is close enough to the pickup loop, and the buffer layer is a non-magnetic layer (like glass)^36^. The scanning SQUID in this approach will measure through the thin support layer (thickness on the order of 10 micrometers).

Lastly, and most importantly, a high throughput version of such microscope should work at room environment, avoiding cooling down the system to reach the SQUID’s operation temperatures. This can be accomplished by using a scanning Diamond NV centers probes^37^ or a scanning SQUID that scans the sample near the vacuum edge of a cryostat, leaving the sample at room temperature^38–40^.

To summarize, we showed a proof-of-concept experiment that demonstrates the potential of integrating scanning SQUID as a readout for microfluidic-based high-throughput screenings. In this example we showed protein interactions tagged by magnetic nanoparticles, using antibodies. Combining scanning SQUID with microfluidics will improve sensitivity and provide a new set of eyes for microfluidic-based readout. Our work aims to motivate the development of a microfluidic-scanning SQUID microscope.

## Methods

### Microfluidic array fabrication

The microfluidic device was fabricated using silicone molds, by casting silicone elastomer polydimethylsiloxane (PDMS, SYLGARD 184, Dow Corning, USA). The device is comprised of two PDMS layers aligned together, and was exposed to chlorotrimethylsilane (TMCS, Sigma-Aldrich) vapor for 10 min to improve elastomer release. For the control and flow molds, two mixtures of elastomer based on silicone and curing agent were prepared in ratios of 5:1 and 20:1, respectively. The control layer was baked and degassed at 80 °C for 30 min. The flow layer was initially spin coated (Laurell, USA) at 2000 rpm for 60 sec and then baked for 30 min at 80 °C. the control channel access holes were punched after the release from the mold. The flow and control layers were then aligned manually under a stereoscope and baked for 2 hours at 80 °C. The two-layer device flow access holes were punched after the release from the mold.

### Fluorescence measurements

We performed fluorescent measurements using microarray scanner (LS Reloaded, Tecan, sensitivity of less than 0.1 Fluorophore equivalent/µm^2^). Alexa Fluor 467 was excited with a laser at 632 nm, and emission signals were collected through a filter at 692 nm ± 25 nm.

### Immunoassay

The target protein (eGFP) was immobilized to the surface through bonding with the epoxy layer. The protein was then detected, using an immunoassay, with a dual fluorescent and magnetic labeled antibody. The detection antibody, Anti-His Alexa Fluor 647 (Qiagen), was conjugated before the experiment to protein G coupled single-core iron oxide nanoparticle with a core diameter of 10 nm (IPG10) (Ocean NanoTech, San Diego, California, USA). To ensure complete coupling between the antibody and IPG10 nanoparticles, an excess (5:1) of nanoparticles was used. We used blocked regions for controls of background noise to establish the scanning SQUID sensitivity. No controls for specificity were used, since specificity is primarily determined by the biochemistry and not by the sensor.

### Preprocessing for magnetic measurements

We used nanoparticles modified by specific antibody against the target protein both on active and control regions to ensure specificity of the desired PPI. Once the reaction reached equilibrium, we dried the solution, leaving behind the magnetic nanoparticles in place. We then removed the microfluidic microarray device that facilitated the surface patterning and experimentation. The latter was required in order to position the SQUID close enough to the surface.

### Scanning SQUID microscopy

For the proof-of-concept experiment described in this manuscript we used a scanning SQUID microscope at cryogenic temperatures^31^ of 4.2 K. We mapped the magnetic landscape several microns above the surface of the chip, with size of 0.9 μm. This limits our ability to separate individual particles in higher concentrations. Scanning area was 350 × 350 µm^2^ with accessible range of 5 × 5 mm^2^. We used a homemade piezo S-benders scanner as described in refs. ^41,42^

## References

[1] Ben-Ari Y (2013). Microfluidic large scale integration of viral–host interaction analysis. Lab Chip.

[2] Venugopal Menon N, Lim SB, Lim CT (2019). Microfluidics for personalized drug screening of cancer. Current Opinion in Pharmacology.

[3] Kool J, Lingeman H, Niessen W, Irth H (2010). High Throughput Screening Methodologies Classified for Major Drug Target Classes According to Target Signaling Pathways. Comb. Chem. High Throughput Screen..

[4] Weng L, Spoonamore JE (2019). Droplet Microfluidics-Enabled High-Throughput Screening for Protein Engineering. Micromachines.

[5] Gerber D, Maerkl SJ, Quake SR (2009). An *in vitro* microfluidic approach to generating protein-interaction networks. Nat. Methods.

[6] Syahir A, Usui K, Tomizaki K-Y, Kajikawa K, Mihara H (2015). Label and Label-Free Detection Techniques for Protein Microarrays. Microarrays (Basel, Switzerland).

[7] Fei X, Gu Y (2009). Progress in modifications and applications of fluorescent dye probe. Prog. Nat. Sci..

[8] Sako Y, Minoghchi S, Yanagida T (2000). Single-molecule imaging of EGFR signalling on the surface of living cells. Nat. Cell Biol..

[9] Axelrod D (2001). Total Internal Reflection Fluorescence Microscopy in Cell Biology. Traffic.

[10] Boozer C, Kim G, Cong S, Guan HW, Londergan T (2006). Looking towards label-free biomolecular interaction analysis in a high-throughput format: a review of new surface plasmon resonance technologies. Current Opinion in Biotechnology.

[11] Wee, K. W. *et al*. Novel electrical detection of label-free disease marker proteins using piezoresistive self-sensing micro-cantilevers. in *Biosensors and Bioelectronics* **20**, 1932–1938 (Elsevier Ltd, 2005). 10.1016/j.bios.2004.09.023 15741060

[12] Vestergaard M, Kerman K, Tamiya E (2007). An Overview of Label-free Electrochemical Protein Sensors. Sensors.

[13] Pleshakova, T. O., Bukharina, N. S., Archakov, A. I. & Ivanov, Y. D. Atomic force microscopy for protein detection and their physicochemical characterization. *International Journal of Molecular Sciences* **19**, (2018). 10.3390/ijms19041142 PMC5979402 29642632

[14] Bruce IJ, Sen T (2005). Surface Modification of Magnetic Nanoparticles with Alkoxysilanes and Their Application in Magnetic Bioseparations. Langmuir.

[15] Mohammed L, Gomaa HG, Ragab D, Zhu J (2017). Magnetic nanoparticles for environmental and biomedical applications: A review. Particuology.

[16] Revia RA, Zhang M (2016). Magnetite nanoparticles for cancer diagnosis, treatment, and treatment monitoring: Recent advances. Materials Today.

[17] Reverté L, Prieto-Simón B, Campàs M (2016). New advances in electrochemical biosensors for the detection of toxins: Nanomaterials, magnetic beads and microfluidics systems. A review. Anal. Chim. Acta.

[18] Tang D, Yuan R, Chai Y (2006). Direct electrochemical immunoassay based on immobilization of protein-magnetic nanoparticle composites on to magnetic electrode surfaces by sterically enhanced magnetic field force. Biotechnol. Lett..

[19] Sokolina K (2017). Systematic protein-protein interaction mapping for clinically relevant human GPCRs. Mol. Syst. Biol..

[20] Wang W (2016). A magnetic nanoparticles relaxation sensor for protein–protein interaction detection at ultra-low magnetic field. Biosens. Bioelectron..

[21] Osterfeld SJ (2008). Multiplex protein assays based on real-time magnetic nanotag sensing. Proc. Natl. Acad. Sci. USA.

[22] Drung D (2007). Highly Sensitive and Easy-to-Use SQUID Sensors. IEEE Trans. Appl. Supercond..

[23] Clarke, J., Braginski, A. I. (Alex I. & John Wiley & Sons). *The SQUID handbook. Vol. 1, Fundamentals and technology of SQUIDs and SQUID systems*. (Wiley-VCH, 2004).

[24] Vrba, J., Nenonen, J. & Trahms, L. Biomagnetism. In *The SQUID Handbook* 269–389 (Wiley-VCH Verlag GmbH & Co. KGaA). 10.1002/9783527609956.ch11 (2006).

[25] Zhang, Y. *et al*. Multi-channel HTS rf SQUID gradiometer system recording fetal and adult magnetocardiograms. In *IEEE Transactions on Applied Superconductivity* **15**, 631–634 (2005).

[26] Hämäläinen M, Hari R, Ilmoniemi RJ, Knuutila J, Lounasmaa OV (1993). Magnetoencephalography theory, instrumentation, and applications to noninvasive studies of the working human brain. Rev. Mod. Phys..

[27] Zhang Y (1993). High temperature RF SQUIDs for biomedical applications. Physiol. Meas..

[28] Oisjoen F (2009). Fast and sensitive measurement of specific antigen-antibody binding reactions with magnetic nanoparticles and HTS SQUID. in. IEEE Transactions on Applied Superconductivity.

[29] Sepehri S (2018). Volume-amplified magnetic bioassay integrated with microfluidic sample handling and high-Tc SQUID magnetic readout. APL Bioeng..

[30] Kirtley JR (1995). High‐resolution scanning SQUID microscope. Appl. Phys. Lett..

[31] Huber ME (2008). Gradiometric micro-SQUID susceptometer for scanning measurements of mesoscopic samples. Rev. Sci. Instrum..

[32] Kirtley, J. R. *et al*. Scanning SQUID susceptometers with sub-micron spatial resolution. *Rev. Sci. Instrum*. **87**, (2016). 10.1063/1.4961982 27782557

[33] Kirtley JR (2009). Prospects for imaging magnetic nanoparticles using a scanning SQUID microscope. Supercond. Sci. Technol..

[34] Yu H (2008). High-Quality Binary Protein Interaction Map of the Yeast Interactome Network. Science (80-.)..

[35] Ketchen, M. B. & Kirtley, J. R. Miniature vector magnetometer for scanning squid microscopy. *IEEE Trans. Appl. Supercond.* **7**, 3139–3142 (1997).

[36] Kalisky B (2013). Locally enhanced conductivity due to the tetragonal domain structure in LaAlO3/SrTiO3 heterointerfaces. Nat. Mater..

[37] Schirhagl R, Chang K, Loretz M, Degen CL (2014). Nitrogen-Vacancy Centers in Diamond: Nanoscale Sensors for Physics and Biology. Annu. Rev. Phys. Chem..

[38] Dechert J, Mueck M, Heiden C (1999). A scanning SQUID microscope for samples at room temperature. IEEE Trans. Appiled Supercond..

[39] Gudoshnikov S (2002). HTS scanning SQUID microscope with high spatial resolution for room temperature samples. Phys. C Supercond..

[40] Lee TS, Chemla YR, Dantsker E, Clarke J (1997). High-Tc SQUID microscope for room temperature samples. IEEE Trans. Appiled Supercond..

[41] Björnsson PG, Gardner BW, Kirtley JR, Moler KA (2001). Scanning superconducting quantum interference device microscope in a dilution refrigerator. Rev. Sci. Instrum..

[42] Shperber, Y. *et al*. Scanning SQUID microscopy in a cryogen-free cooler. *Rev. Sci. Instrum*. **90**, (2019). 10.1063/1.5087060 31153251

